# A Narrative Review of the Association between Dental Abnormalities and Chemotherapy

**DOI:** 10.3390/jcm13164942

**Published:** 2024-08-22

**Authors:** Tatsuya Akitomo, Yasuko Tsuge, Chieko Mitsuhata, Ryota Nomura

**Affiliations:** Department of Pediatric Dentistry, Graduate School of Biomedical and Health Sciences, Hiroshima University, Hiroshima 734-8553, Japan; tsuge08@hiroshima-u.ac.jp (Y.T.); chiekom@hiroshima-u.ac.jp (C.M.); rnomura@hiroshima-u.ac.jp (R.N.)

**Keywords:** dental anomaly, panoramic examination, chemotherapy

## Abstract

Dental abnormalities are often detected in childhood and are reported to occur with high prevalence in patients who have undergone cancer treatment or chemotherapy. We performed a literature search of PubMed from 2004 to 2024 using the terms “dental anomaly” and “panoramic examination”, and 298 potentially relevant articles were found. Thirty-one articles about dental abnormalities matched the eligibility criteria and were extracted for this review. Although the prevalence of tooth agenesis and microdontia in the general population was reported to be approximately 10% and 3%, respectively, the prevalence in patients who had undergone cancer treatment or chemotherapy was higher in all surveys, suggesting that the treatment is related to the occurrence of dental abnormalities. It is important to continue long-term follow-up with patients not only during treatment but also after the completion of treatment. Dental professionals should provide information about dental abnormalities to patients, their guardians, and medical professionals, which may lead to improvement in the quality of life of patients.

## 1. Introduction

Developmental disorders of the teeth, such as abnormal tooth counts and unusual tooth morphology, are often seen in pediatric dentistry [1,2,3,4]. Radiographic and clinical examinations may reveal these dental anomalies [5]. Radiographs are critical for the identification of supernumerary teeth because most are inverted and remain unerupted [6]. Therefore, the radiographic investigation of dental anomalies at an early stage is important [7].

The etiology of these conditions may be a result of genetic factors, etiological events during the prenatal and postnatal development periods, and environmental and pathological factors [5]. Environmental factors such as trauma, infections, radiation, drugs, and hormonal influences have been suggested as possible insults that affect tooth formation during the embryonic stages [8,9].

With the recent progress in the development of chemotherapeutic drugs, the mortality rate from childhood malignancies has declined. However, effects on primary and permanent teeth, such as tooth and root agenesis, root thinning and shortening, and localized enamel defects, may be caused by the administration of chemotherapeutic agents for cancer treatment over a span of years [10,11].

Many studies have investigated the association between dental anomalies and chemotherapy. However, all these studies were limited by small sample sizes, indicating the need for further studies with larger populations [11,12,13]. Therefore, we hypothesized that there may be no real difference in the incidence of dental anomalies between chemotherapy and control groups. As a specific concern, little is known about the detailed incidences of dental abnormalities (including tooth agenesis, hypodontia, and microdonts) caused by chemotherapy and the difference in incidences of dental abnormalities between the chemotherapy and control groups. The aim of this narrative review is to inform medical and dental professionals about the exact frequency of dental anomalies observed in childhood cancer survivors in comparison to control groups and to highlight the importance of oral management when dental anomalies are common in childhood cancer survivors.

## 2. Materials and Methods

### 2.1. Search Strategy

A literature search of PubMed, the electronic database provided by the National Library of Medicine via the Internet (https://pubmed.ncbi.nlm.nih.gov/ (accessed on 29 July 2024)), was conducted by one of the authors on 27 May 2024. Articles were searched manually using the terms “dental anomaly” and “panoramic examination” and filtered to those published from 2004 to 2024.

### 2.2. Inclusion Criteria

The inclusion criteria for this study included the following:

- Articles that could be viewed in their entirety;
- Articles with their full text in English;
- Clinical investigations that were not case reports or reviews;
- Studies that investigated the prevalence of dental abnormalities using panoramic radiography.

### 2.3. Exclusion Criteria

The exclusion criteria for this study included the following:

- Articles that were not suitable for the objective of this review or used the wrong study design;
- Studies that investigated the prevalence of dental abnormalities using cone beam computed tomography imaging;
- Articles about congenital diseases such as syndromes or those with a limited number of subjects or insufficient data; however, a control group was only included when the prevalence of a healthy group was described.

### 2.4. Study Selection

According to the inclusion and exclusion criteria, a literature analysis was performed by two independent examiners (T.A. and Y.T.) to select the articles for this review. Decisions about contentious documents were resolved by discussion.

### 2.5. Data Extraction

The authors extracted the following information: title, authors, year, subject of study, and prevalence of dental abnormalities. We also investigated whether the subjects had undergone cancer treatment or chemotherapy and compared the effects. Data collected were categorized and tabulated according to whether or not cancer treatment or chemotherapy was administered. Dental abnormalities were subdivided into “tooth agenesis or hypodontia” and “microdonts”.

### 2.6. Quality Assessment

After the literature search, the quality of evidence in the articles included in this review was assessed. It was based on a three-level rating scale—low, moderate, and high—with guidance from the Agency for Healthcare Research and Quality [14].

## 3. Results

**Figure 1:**
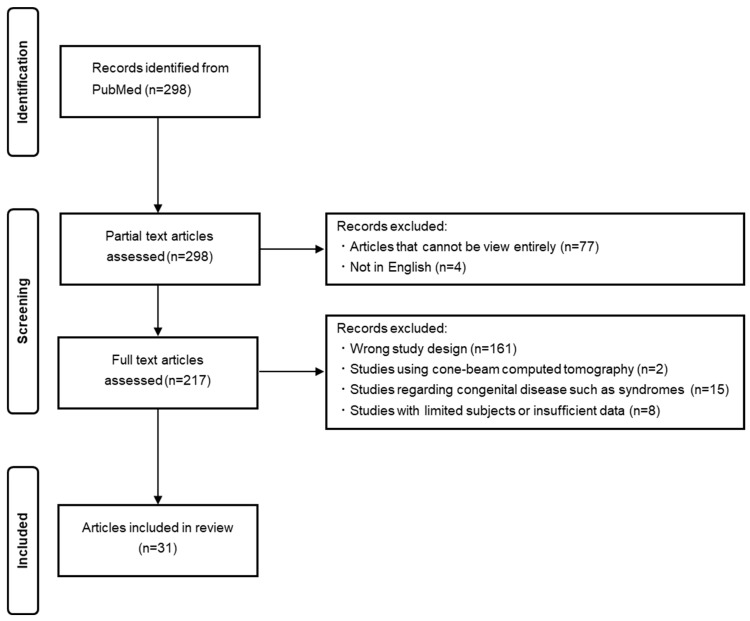
PRISMA flow diagram for the literature search.

Figure 1 shows the PRISMA flow diagram for the literature search. During our literature search, 298 relevant articles were found and 217 articles were selected after partial text article assessment was used to exclude articles that could not be viewed entirely (n = 77) or were not in English (n = 4). After the full-text article assessment of the 217 articles, 31 articles met each criterion and were included in this review. We classified these articles according to whether or not chemotherapy was administered. Articles that investigated the effect of chemotherapy in a chemotherapy group and a control group were included in the tables independently. Of the 31 articles, 27 included information about the prevalence of dental abnormalities in the general population (Table 1) [15,16,17,18,19,20,21,22,23,24,25,26,27,28,29,30,31,32,33,34,35,36,37,38,39,40,41]. Most studies focused on school-age children or teenagers. On the other hand, there were six articles that included patients who had undergone cancer treatment or chemotherapy, and this is shown in Table 2 [22,33,42,43,44,45]. The subjects were school-age children or older, as in the general population.

## 4. Discussion

### 4.1. Dental Anomalies

Dental anomalies are abnormalities in the color, contour, size, and number of teeth, and these anomalies can be related to number, size, shape, and structure [46]. Dental anomalies may occur as a result of factors such as environmental and genetic influences; however, their etiology remains unclear [47,48]. Tooth development is modulated by a specific spatiotemporal molecular pattern of reciprocally inductive epithelium–mesenchyme interactions [49]. Interactions involving positive and negative loops among bone morphogenetic protein, fibroblast growth factors, Sonic hedgehog, and Wnt pathways regulate the morphogenesis of individual teeth [50]. These anomalies can pose complications in the normal functioning of the orofacial complex; however, regular radiographic examination and subsequent correct diagnosis can help prevent complications [51,52]. Therefore, dental professionals should pay attention to the presence of dental abnormalities in regular dental checkups of pediatric patients.

### 4.2. Tooth Agenesis and Hypodontia

Tooth agenesis is the most prevalent craniofacial malformation in humans, and the most commonly affected teeth in the permanent dentition seem to be the mandibular second premolars, followed by the maxillary lateral incisors and the maxillary second premolars [53,54]. Tooth agenesis of one to five teeth is defined as hypodontia, while the absence of six or more teeth is defined as oligodontia [3,55]. Tooth agenesis is common in some syndromes such as Down syndrome, ectodermal dysplasia, and labio-palatal clefts; however, it can also be non-syndromic [56,57,58]. The treatment of a patient with tooth agenesis includes the maintenance of the deciduous teeth, orthodontic treatment for space closure, dental autotransplantation, and prosthodontic treatment such as dental implants or partial dentures. Pediatric dentists, orthodontists, and prosthodontists should work together to achieve optimal long-term treatment outcomes [3,59,60].

### 4.3. Microdonts

A microdont is defined as a tooth that is smaller than normal and smaller than its antimere by more than 1 mm [61]. There are three types of microdontia: true generalized microdontia, relative generalized microdontia (both of which affect the entire dentition), and localized microdontia (which involves only a single tooth) [61,62]. The maxillary lateral incisor is the tooth most commonly affected with microdontia, and it has also been reported in third molars and premolars [63]. Treatment may be required because of esthetic problems and may include orthodontic treatment, restorative treatment, or extraction and tooth replacement [64]. However, Tirone et al. (2016) recommend that treatment should focus on shape adjustment performed with minimally invasive techniques because it is a shape defect [65].

### 4.4. Dental Abnormalities in the General Population

The prevalence of dental abnormalities varied from 1.8 to 75.0 in the general population. Aboujaoude et al. (2023), who reported the highest prevalence of 75%, investigated dental abnormalities, including number, size, shape, position, and structure [37]. Notably, the abnormalities in position included ectopia or impaction. The prevalence may vary because the definitions of dental abnormalities differ in each survey. Therefore, we conducted additional investigations by subdividing dental abnormalities into tooth agenesis and microdontia.

In tooth agenesis, the prevalence of tooth agenesis or hypodontia commonly ranged from 0% to 10%, with one study citing a value of 29.3%. The Japanese Society of Pediatric Dentistry examined more than 15,000 Japanese children and reported a prevalence of tooth agenesis in permanent teeth of 10.09% [66]. The prevalence of microdonts was less than 3% in most studies and there were no differences in the prevalence of microdontia among the results of the studies, suggesting that tooth agenesis occurs in approximately 10% and microdonts occur in 3% of the general population.

### 4.5. Dental Abnormalities in Patients Who Have Undergone Cancer Treatment or Chemotherapy

The prevalence of dental abnormalities ranged from 39.3% to 83.9%. As mentioned above, the prevalence of dental abnormalities depends on the definitions used in each survey. However, even the lowest prevalence among the five articles that recorded detailed figures was 39.3%.

The prevalence of both tooth agenesis or hypodontia and microdonts was approximately 20%, although this varied depending on the report. In addition, all five articles providing detailed figures reported a prevalence of tooth agenesis of over 10%, and three were over 20%. Most of the articles also recorded a prevalence of microdontia of over 20%. These results suggest that patients who have undergone cancer treatment experience dental abnormalities more frequently.

The killing of tumor cells by anticancer therapies commonly used in the treatment of cancer, such as chemotherapy, is predominantly mediated by triggering apoptosis, the cell’s intrinsic death program [67]. Cyclophosphamide (CPA) is one of the most successful drugs for pediatric cancer treatment and is an alkylating agent that inhibits DNA synthesis and produces apoptosis [68,69]. The toxicity of CPA affects not only cancer cells but also normal proliferating tissues, and its severe side effects are associated with the occurrence of cell cycle arrest and apoptosis in several sensitive tissues [68,70]. CPA administration at the early bell stage may disrupt proliferative cells in the tooth germ, resulting in the shrinkage of tooth germ or tooth agenesis in animal experiments; in addition, it is reported that most pediatric cancer survivors receiving CPA treatment develop dental anomalies [68]. Nishimura et al. (2013) reported that the risk of causing tooth formation anomalies with busulfan administration is higher than with cyclophosphamide and that its influence on tooth development is different among alkylators [71]. In addition, bisphosphonates, which are known to treat bisphosphonate-related osteonecrosis of the jaw, have also been reported to cause dental abnormalities [72,73]. The use of drugs in childhood, not just anticancer drugs, may affect tooth formation.

Children with cancer are at high risk of developing nutritional problems related to their underlying disease and side effects of multimodal treatments [74]. In addition, it is reported that prepubertal malnutrition and low body mass in early childhood are associated with dental abnormalities such as enamel defects [75]. On the other hand, Elamin et al. (2013) investigated the effect of severe malnutrition on the timing of human tooth formation, and they concluded that the effect was negligible. This emphasizes the biological stability of the timing of the development of dentition in humans [76]. Therefore, in the future, large-scale studies are needed to elucidate the relationship between dental abnormalities and the type of drugs administered or the nutrition status of the patients.

Kılınç et al. (2019) divided the patients into two groups, Group A (aged 9 months to 4 years at the time of treatment) and Group B (aged 5–7 years at the time of treatment) and reported that the prevalence of tooth agenesis and microdontia was higher in Group A than in Group B [33]. Tanaka et al. (2017) reported that patients aged 0–3 years at the time of treatment had more tooth agenesis or microdontia than those aged 4 years or older [42]. Additionally, several reports found that the younger the age at diagnosis or treatment, the higher the prevalence of dental abnormalities [43,44,45].

Nakatsugawa et al. (2019) reported that the severity of dental anomalies was associated with the developmental stages of the tooth germs at the time of CPA administration, and the cap/early bell stage is the most susceptive timing for tooth agenesis, whereas the late bell stage is affected in root formation [68]. In addition, Hölttä et al. (2005) suggested that it is possible to predict future dental aberrations to some extent by placing the period of therapy on the schedule of tooth mineralization [77]. In fact, although the rate of dental abnormalities was lower in patients aged 4 years or older at treatment, their rate of abnormal root development was higher than in those aged 0–3 years [42]. In our previous study, we analyzed the incidence of abnormal teeth by tooth type and reported that the incidence of tooth agenesis was significantly higher in premolars and second molars, and the incidence of microdonts was significantly higher in premolars than in other teeth [45]. These findings suggest that cancer treatment in childhood causes dental abnormalities at an earlier age; however, the effects vary depending on the time of tooth formation.

### 4.6. Oral Care during Cancer Treatment

Children who undergo chemotherapy and radiation therapy are challenged with the possibility of myriad oral complications including mucositis, xerostomia, and caries [78]. Therefore, comprehensive oral management, including oral care, the removal of dental focal infections, and improvements in oral function with dentures, is conducted for patients with cancer, cardiovascular diseases, and organ transplantation [79].

One of the most frequent complications of chemotherapy is oral mucositis, reported to affect approximately 75% of patients receiving high-dose conditioning chemotherapy before hematopoietic cell transplantation and 20–60% of those being treated for solid tumors [80]. Oral mucositis may decrease the effectiveness of treatment and worsen the quality of life of pediatric oncology patients, and, thus, it is important to reduce the incidence and/or severity of mucositis as much as possible [81]. Cheng et al. (2001) investigated the effectiveness of a preventive oral care protocol and found that the severity of oral mucositis and related pain was significantly reduced [82]. Düzkaya et al. (2017) also reported that the provision of oral care can reduce oral mucositis in children in intensive care [83].

Perioperative oral care intervention is associated with shorter postoperative hospital stays after lung cancer surgeries, and the intervention can prevent the occurrence of postoperative respiratory infections [84]. In addition, Nobuhara et al. (2022) investigated the association between surgical site infection and oral care and concluded that perioperative oral care could reduce the incidence of surgical site infection after colorectal cancer resection [85]. These results indicate that perioperative oral care leads to the prevention of postoperative complications not only in the oral cavity but also in the whole body. The provision of oral care by dental professionals during cancer treatment may reduce complications and improve patient quality of life during hospitalization.

### 4.7. Oral Care after Cancer Treatment

Although survival rates have statistically significantly improved in recent decades, a growing population of pediatric cancer survivors are at risk of long-term therapy-related sequelae [86]. Pediatric cancer survivors may develop enamel demineralization or salivary gland dysfunction, resulting in an increased risk of advanced dental caries [87,88]. Continual dental support leads to the improvement of oral condition and oral health habits; therefore, dental professionals should continue to provide long-term oral care even after patients are discharged from the hospital [89]. However, nearly one-third of pediatric cancer survivors were not seen in a pediatric survivor clinic despite the importance of survivor care, and they may be unaware of their oral and dental risks [90,91]. As reported in this review, pediatric cancer survivors may experience dental abnormalities as a late effect of treatment. Additionally, it is difficult for patients to detect dental abnormalities themselves, and some cases require radiographic examination in a dental clinic. As the health of dentition affects quality of life, pediatric cancer survivors need regular follow-up for the early detection of late effects on dental health [44]. Therefore, it is important to inform patients and their guardians of the risk of dental abnormalities during cancer treatment and to cooperate with medical doctors in the continuation of long-term comprehensive care after discharge.

## 5. Conclusions

This review investigated the prevalence of dental abnormalities in articles published within the past 20 years. The results revealed that the incidence in most of the general population is approximately 0–15% for tooth agenesis and 0–3% for microdontia. In contrast, the incidence in patients undergoing cancer treatment or chemotherapy ranges from 13 to 25% for tooth agenesis and 9 to 65% for microdontia, including a report of extremely high incidence of microdontia. There are limitations in that various factors, such as age at the time of treatment, the stage of tooth formation, the condition of the childhood cancer, treatment drugs, and treatment period, may be involved, and it is difficult to make equal comparisons. In the future, dental professionals will need to report more studies regarding the follow-up of pediatric cancer survivors and increase their evidence. This review highlights that the incidence of dental abnormalities is higher in patients who have undergone chemotherapy, emphasizing the importance of oral care not only during the chemotherapy treatment but also after the completion of the treatment.

## Figures and Tables

**Table 1:** Studies selected for the review—general population.

Author	Quality of Evidence	Subject	Mean Age [Years (Range)]	Dental Abnormalities (%)	Tooth Agenesis or Hypodontia (%)	Microdonts (%)
Lexner MO, 2007 [15]	Low	Female controls (n = 73)	29 * (15–58)	-	9 *	-
Chung CJ, 2008 [16]	High	Patients with mixed to permanent dentition (n = 1622)	-	-	11.2	-
Paulsson L, 2008 [17]	Low	Control group with full-term normal birth weight (n = 41)	9.5	-	4.9	-
Abe R, 2010 [18]	Low	Control group (n = 32)	8.8	-	6.3	-
Ajami BA, 2010 [19]	Moderate	Children aged 9–14 years (n = 600)	10.6	-	9.0	-
Gupta SK, 2011 [20]	Moderate	Indian subjects (n = 1123)	-	34.3	4.5	2.6
Kim YH, 2011 [21]	High	Korean orthodontic patients (n = 3055)	15.1 (9–30)	-	11.3	-
Lauritano D, 2012 [22]	Low	Control group (n = 52)	11.0	-	3.8	7.6
Mukhopadhyay S, 2014 [23]	Low	Bengali-speaking nursery children (n = 2757)	- (4–6)	1.8	0.5	-
Fekonja A, 2015 [24]	Moderate	Caucasian subjects born in 1966, 1976, 1986, and 1996 (n = 2546)	-	11.5	6.9	-
Medina AC, 2016 [25]	Low	Healthy Venezuelan children (n = 1188)	- (5–12)	-	5.6	-
Yassin SM, 2016 [26]	Low	Saudi children (n = 1252)	- (5–12)	25.4	9.7	2.6
Fekonja A, 2017 [27]	Moderate	Patients treated at the orthodontic department (n = 473)	14.2	16.7	7.2	2.5
Gracco ALT, 2017 [28]	High	Caucasian orthodontic patients (n = 4006)	- (9–16)	-	9.0	-
Park MK, 2017 [29]	Moderate	Patients with no systemic diseases (n = 4611)	- (6.0–12.9)	-	9.1	-
Dallel I, 2018 [30]	Moderate	Orthodontic patients (n = 1000)	20 *	-	7.8	-
Fernandez CCA, 2018 [31]	Moderate	Orthodontic records of patients (n = 1047)	16.4	15.7	9.7	5.3
Septer S, 2018 [32]	Low	Control group (n = 46)	13.7	54.3	6.5	-
Kılınç G, 2019 [33]	Low	Healthy siblings of pediatric patients treated for cancer (n = 93)	10.6 (8–16)	9.7	0.0	0.0
MacDonald D, 2020 [34]	High	New patient examination (n = 6252)	- (7–90)	32.1	1.5	-
Wagner VP, 2020 [35]	Moderate	Patients who received panoramic examination 2014–2016 (n = 512)	8.8 (6–12)	61.3	29.3	0.6
Jankowski T, 2021 [36]	Low	Control group (n = 100)	10.3 (6–15)	3.0	1.0	-
Aboujaoude S, 2023 [37]	Low	Lebanese children (n = 112)	10.5 (8–15)	75.0	16.1	-
AlHudaithi FS, 2023 [38]	Moderate	Orthodontic patients (n = 384)	- (12–)	-	7.3	-
Alanzi A, 2024 [39]	Moderate	Pediatric patients (n = 586)	- (8–12)	20.1	9.3	0.5
Matošić Ž, 2024 [40]	Moderate	Female control group (n = 424)	- (20–40)	-	3.5	0.9
Mohan R, 2024 [41]	Moderate	Non-syndromic pediatric patients (n = 581)	- (6–18)	73.8	15.1	1.0

* The first decimal place was not described. The hyphen means the information was not described.

**Table 2:** Studies selected for the review—patients who had undergone cancer treatment or chemotherapy.

Author	Quality of Evidence	Subject	Mean Age [Years (Range)]	Dental Abnormalities (%)	Tooth Agenesis or Hypodontia (%)	Microdonts (%)
Lauritano D, 2012 [22]	Moderate	Children in long-term remission after leukemia treatment (n = 52)	11.5	-	13.4	23.1
Tanaka M, 2017 [42]	Low	Childhood cancer survivors (n = 56)	13.9 (4.6–32.7)	46.4	16.1	21.4
Kang CM, 2018 [43]	Moderate	Childhood cancer survivors (n = 196)	15.6	55.6	20.4	30.6
Kılınç G, 2019 [33]	Moderate	Pediatric patients diagnosed with cancer and treated (n = 93)	9.5 (8–13)	83.9	22.6	64.5
Immonen E, 2021 [44]	Moderate	Leukemia survivors (n = 178)	5.0	39.3	-	-
Akitomo T, 2024 [45]	Moderate	Patients who received chemotherapy (n = 32)	- (7–)	46.9	25.0	9.4

The hyphen means the information was not discribed.

## Data Availability

The data are available from the corresponding author upon reasonable request.
